# Parental socioeconomic position, childhood poverty and healthy aging: retrospective and prospective study of six cohorts across 32 wealthy and developing nations

**DOI:** 10.3389/fpubh.2026.1879570

**Published:** 2026-08-10

**Authors:** Gindo Tampubolon

**Affiliations:** 1 United Kingdom National Institute for Health and Care Research (UK NIHR) Policy Research Unit on Healthy Aging, University of Manchester, Manchester, United Kingdom; 2 Global Development Institute, University of Manchester, Manchester, United Kingdom

**Keywords:** CHARLS, childhood poverty, ELSA, healthy aging scale, HRS, IFLS, international comparison, latent trait

## Abstract

**Background and aim:**

Children who were poor in wealthy countries have reported worse cognitive, muscle, and mental functions, as well as greater frailty and multimorbidity, in later life. However, it remains unclear whether those who were poor in childhood worldwide fall short of healthy aging, as information on childhood conditions is often unreliable. Here, I present new evidence on the lifecourse shaping of healthy aging among older adults worldwide.

**Methods:**

Some 80,000 older adults aged 50 and over in 32 wealthy and developing nations recalled their childhood conditions at ages 10–14 and then prospectively reported their health functioning. By the decade's end, these countries will host more than half (53%) of the world population aged 60 years and over, according to the United Nations. Following recent empirical studies, a modified healthy aging scale is constructed using a generalized latent trait model. Childhood conditions in England and Europe include the number of books, rooms, and people (indicating overcrowding), the presence of running hot water, and the presence of central heating. Across America, these are mostly replaced with financial hardship or family indebtedness; in China, starvation to death due to government edict; and in Indonesia, the presence of running cold water. As in prior practice, childhood poverty is treated as a latent construct underlying these error-prone recollections. Its associations with the modified healthy aging scale are estimated using country fixed-effects regression, controlling for parental socioeconomic position, age, sex, education, wealth, and marital status. Extensive sensitivity analyses assess robustness.

**Results and discussion:**

Childhood poverty is associated with reduced healthy aging by 0.14 standard deviations (*z* = −17.6); older adults who grew up poor have lower healthy aging scores than those who were not poor. Women reported lower healthy aging scores. Distributions of healthy aging vary across countries, as do age profiles of healthy aging among older adults. Childhood recollections show that those who were poor in childhood enter later life with poorer healthy aging, making the life course shaping of healthy aging central. The strong evidence presented here calls for urgent action to eliminate child poverty, given its lifelong rewards. Researchers can now use childhood recollections from wealthy and developing nations alike to estimate associations with older adults' health. Health policymakers should adopt a life course approach when evaluating childhood health interventions, given their lifelong promise.

## 1 Introduction

The 21st century began with an unprecedented demographic shift: population aging is accelerating worldwide, posing ongoing challenges for society, states and healthcare systems. The scale and speed of aging in developing nations pose a concentrated challenge; what took France a century will take China only a few decades, according to the World Health Organization (1). The WHO has thus designated 2021–2030 as the UN Decade of Healthy Aging, heralding a global commitment to foster healthier, longer lives (2, 3). This requires a fundamental re-evaluation of how health is promoted across the human life course.

Health measures have often been limited to specific populations and have emphasized the absence of disease. Fortunately, a more fruitful understanding of health in the context of population aging has emerged. The WHO defines healthy aging as “the process of developing and maintaining the functional ability that enables wellbeing in older age” (1, 4). This functional ability is dynamically shaped by the interaction between a person's intrinsic capacity and the environment, including physical and psychological faculties such as vitality, locomotion, and cognitive, mental, and sensory functions (1, 5).

Empirical research on healthy aging has largely followed two approaches, as exemplified by two 2021 studies: the healthy aging scale (6) (using a latent trait approach) and the healthy aging index (7) (using a counting approach) (8). Although the approaches differ, both constructs aim to capture the biopsychosocial aspects of health and functioning, integrating domains of vitality, sensory functions, locomotion, cognition, and activities of daily living that reflect a person's interaction with the environment (though the healthy aging scale conspicuously omits mental health items in its construction) (6, 7). This multidimensional approach is fundamental because social engagement and mental capacities are as important as biological factors for achieving healthy aging (1–3, 5, 9).

### 1.1 Two life course frameworks

A critical instrument in explaining healthy aging is the life course framework of health which has been receiving mounting empirical support (10–17). This posits that health outcomes are not exclusively determined by current conditions but are shaped by a complex interplay of protective and risk factors throughout life, from before birth to old age. This temporal and societal perspective emphasizes that investments in health at any life stage yield compounded benefits over time and for subsequent generations, reminding us that early factors can shape physical and mental capacities in later life, including muscle and cognitive function and mental health (10, 13, 14). Critical and sensitive periods (e.g., early childhood, adolescence, mid-life) and significant transition periods (e.g., entering school, parenthood, retirement) are recognized as pivotal moments where exposures can have profound and lasting effects on health trajectories.

**Figure 1:**
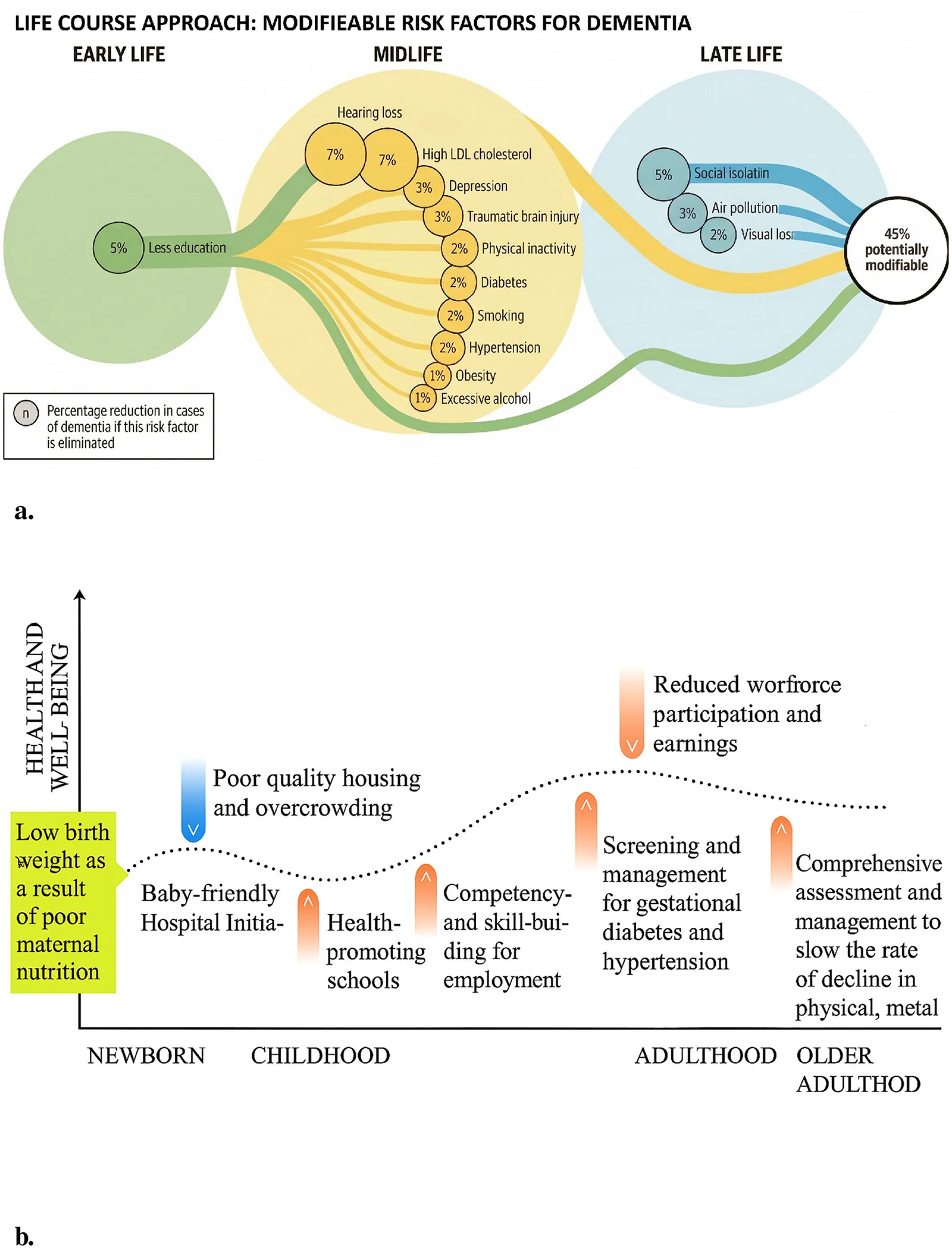
Two life course frameworks. **(a)** Redrawn from the Lancet Commission on Dementia (2024). **(b)** WHO Framework to implement a life course approach in practice (2025). Adapted from World Health Organization (2025); Livingston, et al. (2024).

Here are two recent frameworks (Figure 1) on the life course shaping of older adults' health (first, dementia from the Lancet Commission, and second, broader outcomes from the WHO) (5, 18). Compared with the WHO framework, the Lancet Commission framework appears less detailed at the childhood stage and is therefore used here to guide research highlighting the role of overcrowding and housing quality. These specific features have been amply shown to shape older adults' health across up to 31 wealthy and developing nations in single- and cross-country studies, explaining gait speed, depression, episodic memory, probable sarcopenia, frailty, and multimorbidity (13–15, 19).

Evidence has repeatedly shown that childhood poverty is a risk factor for disability, dysfunction, and disease in later life across wealthy and developing nations, including the US, Britain, China, and Europe. A recent study significantly expanded this understanding, presenting the first analysis across 31 diverse countries demonstrating that childhood poverty is a strong predictor of multimorbidity, or multiple chronic conditions, in later life (19, 20). This association, with childhood poverty linked to a higher probability of multimorbidity, holds across varied health systems, from the UK National Health Service to largely private systems in the US. The study draws on five major longitudinal cohorts—the US Health & Retirement Study (HRS), the English Longitudinal Study of Aging (ELSA), the Survey of Health Aging & Retirement in Europe (SHARE), the China Health and Retirement Longitudinal Study (CHARLS), and the Indonesia Family Life Survey (IFLS) – encompassing a broad spectrum of aging experiences across different levels of socioeconomic development.

In the study, we have suggested a potential epigenetic explanation, supported by evidence from the US, where childhood poverty is associated with more adverse epigenetic changes, as measured by increased methylation rates in old age (14, 21). This extensive geographic reach and the diverse health systems under examination suggest that the life course shaping of healthy aging hypothesis can be usefully examined elsewhere.

Obtaining such evidence also highlighted methodological challenges in applying the life course framework, especially when it relies on retrospective information about childhood conditions from several decades ago. I found, for instance (13),

In 2008, some 2,500 Britons aged 50 were asked to recall the number of rooms and people in the house when they were 11, to assess overcrowding. Only one in three got both numbers right (compared with their mothers' original answers from the earlier visit).

The same cohort, i.e., the 1958 National Child Development Study, were again visited when aged 62 in 2020 (with some inevitable attrition in between)—I found only 36% got both numbers right (22–24). Across the oceans, in Indonesia in 2015 I found fewer than one in ten older adults got both numbers right (19). Retrospective information of childhood conditions is mostly erroneous when elicited from older adults in both wealthy and developing nations. Yet, most empirical studies use the information as error-free.

In the same article (13), we therefore presented a theoretical justification for a latent construct of childhood poverty that addresses this considerable problem of erroneous recall in implementing a life course approach. Childhood material lack shapes older adults' health through both direct and indirect pathways via constrained educational and occupational trajectories, implying that early-life conditions exert enduring, multiple influences across the life course rather than operating through a single channel. However, empirical information on childhood material conditions in ongoing aging cohorts relies heavily on erroneous recall. Specifying childhood material poverty as a latent construct allows researchers to use available retrospective indicators (often imperfect) while explicitly accounting for measurement error, thereby more effectively capturing the underlying life course exposure that shapes healthy aging. Unsurprisingly, available childhood indicators vary across aging cohorts, arising from differences in history (for instance, mass starvation is unique) or in latitude (from the equator to the sub-arctic). Fortunately, in recent studies, the use of a latent construct of childhood poverty has been found effective and essential to handle erroneous recall when researching the life course shaping of health (12–15, 19).

Beyond childhood poverty, broader socio-economic inequalities consistently influence healthy aging across nations. Research comparing the US, England, China, and Japan shows that older people with disadvantaged socio-economic positions are less likely to achieve healthy aging than their peers with advantaged positions (7). This multinational comparison, which also drew from the HRS, ELSA, CHARLS, and the Japanese Study of Aging and Retirement, demonstrated that education was an influential socio-economic predictor of healthy aging. This highlights the influence of differing national contexts and health systems; for instance, Japan, with its low-cost health services, exhibits relatively small socioeconomic inequality in healthy aging and generally the best healthy aging profile among the nations studied (7). Conversely, the educational inequality in healthy aging in China was distinctly larger than any other socioeconomic inequality, likely reflecting the historical and economic transformations that have deeply stratified educational opportunities and subsequent health outcomes (7). The accumulating evidence paints a clear picture: what happens in early life profoundly shapes health in later life.

Despite the growing body of evidence, leveraging these insights to inform policy and practice across the diverse landscapes of wealthy and developing nations presents complex challenges, including errors in the recall of retrospective information and the harmonization of information across nations. Further research is needed to refine measurement tools, design effective interventions, and harness the power of longitudinal aging surveys for effective global health policy.

### 1.2 Healthy aging and its measurement: healthy aging index and healthy aging scale

The WHO defines healthy aging positively as the “process of developing and maintaining the functional ability that enables wellbeing in older age,” placing functional ability at the center of research and policy. Such functional ability arises from the interaction between a person's intrinsic capacity (a multidimensional and latent construct) and the environments in which one lives. Healthy aging therefore reflects both personal resources and the opportunities and constraints created by social, economic, and physical environments (1, 4). Healthy aging is thus best understood as the cumulative product of biological, behavioral, and social processes unfolding across the life course. This conceptualization has important implications for measurement: indicators of healthy aging should capture multiple dimensions of functioning rather than relying solely on disease counts or mortality, thereby reflecting the broader goal of maintaining wellbeing, participation, and independence in older age, all positive qualities of the aging experience.

To deal with the challenges, this study stands on the shoulders of two recent multinational studies on the healthy aging scale (6) and the healthy aging index (7), and it is closest in purpose to a most recent study by Wu and colleagues (ATHLOS project), which also used the healthy aging scale (25). In comparison, this study differs along two key dimensions: the exposure of childhood poverty and the outcome construction, i.e., the healthy aging scale. Unlike in the most recent study which used parental occupation and education (25), in a series of recent multinational studies on the life course shaping of health in later life (12, 14, 15, 19), the exposure of early life adversity is a latent construct with multiple items or indicators including housing quality and overcrowding—indicators that are singled out in the WHO Framework to implement a life course approach (5).

Over the last couple of decades, measures of healthy aging have not settled to a consensus, but it has generally been measured in two main approaches. First, by measuring multiple organ functions and comparing them with clinical cut-offs, then summing the scores or counting the affirmative comparisons. There may be variations in how comparisons are made, but essentially this counting approach relies directly on observed measures. See, for example, the healthy aging phenotype and more recently the healthy aging index (7, 8, 26). Conversely, a recent latent approach, exemplified in a couple of studies from the ATHLOS project and the Health and Retirement Study, relies on latent constructs. For example, the ATHLOS project applied a latent trait model (formulated in 1968) (27) to 41 observed measurements when deriving the latent trait of healthy aging. The trait parameter is interpreted as the healthy aging scale, with higher means indicating healthier. Notably, ATHLOS' healthy aging scale does not include mental health, while the healthy aging index does.

To avoid yet another measure of healthy aging, this study closely follows the latent approach of ATHLOS by applying a more recent generalized latent trait model (proposed in 2000) (28); this time, it includes mental health functions, an important condition in late life (13, 14, 29). Because the study aims not to derive a scale of healthy aging but to estimate the strength of association between healthy aging and childhood poverty under a wide variety of national circumstances, I follow the prior practice of focusing on the association. Courtin and colleagues, for example, estimated the association between mental health and socioeconomic position in older adults over 50 years, comparing the US (HRS) and Europe (SHARE) (30). Even though HRS and SHARE use different instruments (Center for Epidemiologic Studies–Depression and Euro-Depression scales), resulting in important differences in the prevalence of depression cases, the authors nevertheless proceeded with the estimation of the association between mental health and socioeconomic position. Similarly, Tampubolon and Maharani estimated the association between allostatic load trajectories and their risk factors in the US (HRS) and England (ELSA) despite the two longitudinal cohorts collecting different blood biomarkers (31), In sum, my focus is not on the difference in averages of the healthy aging scale across nations but on the strengths of its associations with childhood poverty.

### 1.3 A new healthy aging scale: from latent trait model to generalized latent trait model

Because the healthy aging scale has been used in many countries and the latent trait (healthy aging) is useful, I start with the healthy aging scale and proceed with two modifications and call the result healthy aging scale (modified) (12–17, 20, 32, 33). First, I include an item on mental health, specifically CESD and Euro-D. The second and more important advance concerns the latent construction. The healthy aging scale (original) proceeded by dichotomizing all continuous items at the first quartile (6) to make the items fit the latent trait model of Birnbaum (27). This practice has long been suspect—it is well-known that dichotomizing continuous variables leads to loss of information and inconsistent estimation (34–37). Table 1 below shows the number of items that are exposed to such risks.

**Table 1:** Items for healthy aging scale (6) and for healthy aging index (7).

Domains	Items for healthy aging scale	Items for healthy aging index
Cognition	Memory	
	Immediate recall	*
	Delayed recall	*
	Verbal fluency	
	Orientation in time	*
	Processing speed	
	Numeracy	
Psychology	Sleeping	Life satisfaction
		CES-D, Euro-D
Vitality	Experiences some degree of pain	
	Having a high level of energy	
	Urinary incontinence	
Sensory function	Near vision	
	Far vision	
	Eyesight using glasses or lens as usual	
	Hearing in general	
	Hearing in a conversation	
Loco/mobility	Stooping, kneeling or crouching	*
	Lifting or carrying weights	*
	Climbing stairs	*
	Getting up from sitting down	*
	Walking by yourself and without any equipment Pulling or pushing large objects	
	Sitting for long periods	
	Reaching or extending arms	*
	Walking speed	
	Dizziness when walking on a level surface	
	Picking up things with fingers	*
Grip strength		Grip strength
ADL	Getting in or out of bed	*
	Bathing or showering	*
	Getting dressed	*
	Moving around the home	*
	Using the toilet	*
	Eating	*
IADL	Doing housework	
	Shopping for groceries	
	Getting out of the house	
	Difficulties in preparing meals	
	Using map	
	Managing money, bills or expenses	
	Taking medications	
	Making telephone calls	

* Same as the left column.

This is doubly acute for multinational studies like this (32 nations) because variation in a continuous variable, e.g., depression, is likely considerable across countries. For example, with the cut-off at four to mark “case” from “non-case” (using the Euro-D instrument), yielding a binary variable of depression—a chi-square test of the difference in depression rates between Swedish and Danish Men shows no significant difference ($\chi_{1}^{2}=2.46,p=0.12$). But a *t* test of the original Euro-D scores shows a highly significant difference in means (|*z*| = 2.677, *p* = 0.007).

Already in 1983 Cohen wrote, “since methods are available for making use of all the original scale information there is no reason to sustain [dichotomizing practice]” (34). The warning is regular (35, 37) and more recently MacCallum and colleagues wrote, “[dichotomizing practice] is rarely defensible and often will yield misleading results” (36). There is another reason to use a more modern generalized latent trait model—a direct descendant of Birnbaum's 1968 latent trait model—it is capable of ingesting all scale information, not only dichotomous or binary (28). The generalized latent trait model has been available since 2000; for details and comparisons see Birnbaum (27) and Moustaki and Knott (28). I use this generalization to derive a healthy aging scale (modified) with a similar set of items except for the addition of mental health.

In sum, the construction of a modified healthy aging scale is guided by two principles. First, pragmatism (6, 7, 38), so I start with solid bases (healthy aging scale and healthy aging index). Second, I apply recent methodological developments (28, 39–44). The items for constructing the scale are shown in Table 1.

### 1.4 Aims

I aim to explore the associations between childhood poverty and the healthy aging scale within the new WHO Life Course Framework, comparing experiences across wealthy and developing nations, not least because findings on healthy aging have been deeply unbalanced between these nations (4, 45). This raises the following questions:

- Does childhood poverty directly associate with healthy aging in wealthy and developing nations even after parental and adulthood risk factors are considered?
- How can ongoing longitudinal cohort studies be used to better capture variations in healthy aging across diverse global populations?

## 2 Materials and methods

The outcome is the healthy aging scale (modified), which is based on ATHLOS' healthy aging scale with two modifications (6). First, it includes a depression scale (CESD or EuroD) for the psychology domain; second, it replaces Birnbaum's latent trait model (1968) with Moustaki and Knott's generalized latent trait model (2000), which relaxes the items' metric (27, 28). Unlike Birnbaum's model, the generalized model admits all metrics: binary, ordered, and continuous of various distributions. The depression scale, for instance, has a gamma distribution. *For each cohort, the model is fitted to derive a latent trait* which, following the ATHLOS project (6), is called the Healthy Aging Scale (modified) with mean zero and variance one.

The key exposure is childhood poverty, a latent construct of error-prone childhood information collected in life history interviews, following ample precedents (12–17, 19, 20, 46). It is twofold: poor, non-poor. The childhood information or indicators are presented in Table 2, again following prior practice (13–15, 19, 47). They include, among others, overcrowding, lack of home facilities such as indoor toilet, number of books; these are related to the latent class of childhood poverty as specified in a standard latent class model (12–15, 19, 48–51).

**Table 2:** Retrospective childhood information in HRS, ELSA, SHARE, TILDA, CHARLS, and IFLS.

Surveys	HRS and TILDA (age ≤ 16)	ELSA and SHARE (age 10)
Variable	Number of rooms	Number of rooms
	Number of people	Number of people
	Number of books	Number of books
	Ever experienced: being financially poor, had to move or be helped because of financial difficulty, had to live with grandparents, father unemployed	Physical facilities: indoor toilet, fixed bath, central heating, hot & cold running water
Surveys	CHARLS (age ≤ 17)	IFLS (age 12)
Variable	Family member starved to death	Number of rooms
	Had to move due to financial difficulty	Number of people
	Experienced financial hardship	Had electricity
	Living in commune that is unsafe	Had cold running water
	…less willing to help others	Had indoor toilet
	…not close knit	Experienced hunger or famine
	…not clean	

The two-class solution was based on prior literature, which in turn considered fit statistics [Akaike and Bayes-Schwarz information criteria (AIC, BIC)], parsimony, and ease of interpretation (12–15, 19, 50, 51). Unfortunately, the set of items or indicators varies across cohorts. Balanced against this is the affordance of large-scale cross-national study even if the sets are not uniform. *For each cohort, the latent class model is fitted to derive childhood poverty*.

The other controls include parental socioeconomic or occupational position (twofold: low, high) following prior practice (14, 15, 19, 20, 25, 52). A set of confounders or social determinants for adjustment is included as standard in the empirical literature on aging including age, sex, education, wealth, marital status and country (13–15, 53, 54). The resulting Healthy Aging Scale (modified) and childhood poverty and the controls are then pooled, retaining the 32 country indicators. I then fit a country fixed-effects regression to this analytic sample, estimating 31 country dummies. As part of a sensitivity analysis, a more parsimonious model, random intercepts regression, is fitted, which obviates the need to estimate the 31 extra coefficients by assuming the countries are drawn from a super-population of countries. Significance is set at 5% and all estimation is done with Latent Gold syntax 6.1 (55–57).

## 3 Results

### 3.1 Summary statistics of the analytic sample

The analytic sample comprises 80,319 adults aged 50+ drawn from six ongoing longitudinal aging cohorts (HRS, ELSA, SHARE, TILDA, CHARLS, IFLS), with a balanced age profile (mean age 65.6 years, SD 9.5 years) and a modest women majority (45,476 women; 56.6%). Life course exposure of interest (childhood poverty) is common: nearly one in four participants are classified as childhood poor (24.5%), with a higher prevalence among women (25.2%) than men (23.6%). At baseline, the Healthy Aging Scale (HAS, modified) shows a small advantage for men (men: mean 0.2; women: –0.1), consistent with sex differences observed in functional and mobility domains in many cohorts.

Educational attainment is heterogeneous across nations, but in aggregate, 40.4% report up to primary education, 37.8% have completed high school, and 21.8% have a college education or higher. This gradient presents the sizeable education coefficients in multivariable models and mirrors cross-national disparities in access to schooling among the older cohorts under study. Current socioeconomic position (wealth quartiles) remains strongly skewed: 19.7% of the sample are in the bottom wealth quartile, with a larger concentration among women (21.8% vs. 17.0% in men). The intergenerational association is captured by parental socioeconomic/occupational position, where roughly 30% report low parental SEP -consistent with post-war and transitional-economy cohorts.

Marital composition varies by sex: men are more often married/partnered (75.7% vs. 60.1%), whereas women are more often separated/widowed (35.4% vs. 18.8%), a familiar pattern reflecting sex differences in longevity. Geographically, Europe supplies the bulk of observations (via SHARE and TILDA), with notable contributions from the USA (8.0% of the sample), China (11.7%) and Indonesia (3.9%). Ireland and England together account for 14.4% via TILDA and ELSA. The spread across 32 nations ensures ample between-country variation in social histories (e.g., welfare regimes, rapid development) and health systems (public, mixed, private), which is then absorbed in fixed-effects estimation.

Altogether, Table 3 indicates (i) substantial heterogeneity in socioeconomic conditions across the life course, (ii) a non-trivial burden of childhood poverty, and (iii) sex, education, and wealth patterns that are plausibly linked to healthy aging. These features provide statistical power and external validity for modeling associations between childhood poverty and later-life healthy aging while underscoring the need to adjust for contemporaneous socioeconomic factors and parental socioeconomic position.

**Table 3:** Summary statistics of analytic sample for explaining the healthy aging scale drawn from six longitudinal aging cohorts (HRS, ELSA, SHARE, TILDA, CHARLS, IFLS).

	Men	Women	Total
	*N* = 34, 843	*N* = 45, 476	*N* = 80, 319
Childhood poor			
Childhood non-poor	26,625 (76.4%)	34,027 (74.8%)	60,652 (75.5%)
Childhood poor	8,218 (23.6%)	11,449 (25.2%)	19,667 (24.5%)
Healthy aging scale	0.2 (1.0)	−0.1 (1.0)	−0.0 (1.0)
Age	65.7 (9.2)	65.5 (9.7)	65.6 (9.5)
Education			
Up to Primary	12,574 (36.1%)	19,852 (43.7%)	32,426 (40.4%)
High school	14,059 (40.3%)	16,292 (35.8%)	30,351 (37.8%)
College/more	8,210 (23.6%)	9,332 (20.5%)	17,542 (21.8%)
Marital status			
Single	1,902 (5.5%)	2,056 (4.5%)	3,958 (4.9%)
Married/ Partnered	26,385 (75.7%)	27,315 (60.1%)	53,700 (66.9%)
Sep/Widowed	6,556 (18.8%)	16,105 (35.4%)	22,661 (28.2%)
Current wealth			
Above bottom quartile	28,935 (83.0%)	35,568 (78.2%)	64,503 (80.3%)
Bottom quartile	5,908 (17.0%)	9,908 (21.8%)	15,816 (19.7%)
Parent socioeconomic position			
SEP high	24,121 (69.2%)	31,805 (69.9%)	55,926 (69.6%)
SEP low	10,722 (30.8%)	13,671 (30.1%)	24,393 (30.4%)
Country			
Ireland	1,965 (5.6%)	2,351 (5.2%)	4,316 (5.4%)
England	3,207 (9.2%)	4,019 (8.8%)	7,226 (9.0%)
Austria	742 (2.1%)	1,128 (2.5%)	1,870 (2.3%)
Germany	1,660 (4.8%)	1,864 (4.1%)	3,524 (4.4%)
Sweden	1,245 (3.6%)	1,459 (3.2%)	2,704 (3.4%)
Netherlands	644 (1.8%)	842 (1.9%)	1,486 (1.9%)
Spain	1,254 (3.6%)	1,547 (3.4%)	2,801 (3.5%)
Italy	1,415 (4.1%)	1,737 (3.8%)	3,152 (3.9%)
France	1,351 (3.9%)	1,843 (4.1%)	3,194 (4.0%)
Denmark	1,294 (3.7%)	1,499 (3.3%)	2,793 (3.5%)
Greece	332 (1.0%)	431 (0.9%)	763 (0.9%)
Switzerland	1,024 (2.9%)	1,244 (2.7%)	2,268 (2.8%)
Belgium	1,499 (4.3%)	1,827 (4.0%)	3,326 (4.1%)
Israel	333 (1.0%)	486 (1.1%)	819 (1.0%)
Czechia	1,216 (3.5%)	1,891 (4.2%)	3,107 (3.9%)
Poland	1,253 (3.6%)	1,578 (3.5%)	2,831 (3.5%)
Luxembourg	377 (1.1%)	461 (1.0%)	838 (1.0%)
Hungary	262 (0.8%)	413 (0.9%)	675 (0.8%)
Slovenia	937 (2.7%)	1,356 (3.0%)	2,293 (2.9%)
Estonia	1,031 (3.0%)	1,825 (4.0%)	2,856 (3.6%)
Croatia	493 (1.4%)	635 (1.4%)	1,128 (1.4%)
Lithuania	475 (1.4%)	861 (1.9%)	1,336 (1.7%)
Bulgaria	348 (1.0%)	520 (1.1%)	868 (1.1%)
Cyprus	182 (0.5%)	311 (0.7%)	493 (0.6%)
Finland	520 (1.5%)	592 (1.3%)	1,112 (1.4%)
Latvia	267 (0.8%)	470 (1.0%)	737 (0.9%)
Malta	335 (1.0%)	425 (0.9%)	760 (0.9%)
Romania	513 (1.5%)	694 (1.5%)	1,207 (1.5%)
Slovakia	431 (1.2%)	508 (1.1%)	939 (1.2%)
USA	2,527 (7.3%)	3,859 (8.5%)	6,386 (8.0%)
Indonesia	1,234 (3.5%)	1,863 (4.1%)	3,097 (3.9%)
China	4,477 (12.8%)	4,937 (10.9%)	9,414 (11.7%)

### 3.2 Model results for the healthy aging scale

Both fixed-effects and random-effects (Table 4) specifications return nearly identical coefficients, indicating that the main inferences are robust to alternative assumptions about cross-country heterogeneity. The coefficient for childhood poverty is –0.14 (*z* = 17.6), implying that, net of covariates, older adults who grew up poor have healthy aging scores that are 0.14 standard deviations (SD) lower than those who were not poor. This is a meaningful gap: it is roughly half the magnitude of the women–men difference and a quarter of the college advantage. Sex differences are pronounced: women score –0.276 SD lower than men, all else equal. Some of this may reflect differential exposure to caregiving, for instance; the models control for marital status and wealth, strongly suggesting that additional pathways (e.g., unpaid care histories) may be at play.

**Table 4:** Results of model estimation for healthy aging scale: country fixed effects regression and random intercepts regression.

	Country fixed effects regression	Random intercepts regression
Childhood poor	−0.140^***^	−0.140^***^
	(−17.54)	(−17.68)
Sex, Women	−0.276^***^	−0.276^***^
	(−45.01)	(−45.01)
Age	−0.038^***^	−0.038^***^
	(−109.83)	(−109.86)
High school	0.294^***^	0.294^***^
	(38.93)	(39.03)
College/more	0.534^***^	0.535^***^
	(58.86)	(58.98)
Married/Partnered	0.153^***^	0.153^***^
	(10.88)	(10.85)
Separated/Widowed	0.088^***^	0.087^***^
	(5.96)	(5.94)
Bottom quartile wealth	−0.255^***^	−0.256^***^
	(−29.35)	(−29.59)
Parents' occupational position low	−0.050^***^	−0.051^***^
	(−6.16)	(−6.25)
Constant	2.388^***^	2.379^***^
	(83.79)	(66.92)
Country dummies	Yes	No
Observations	80,313	80,313
Adjusted *R*^2^	0.283	0.269

*t* statistics in parentheses; ^*^p < 0.1, ^**^p < 0.05, ^***^p < 0.01.

Age has the expected negative slope (–0.038 per year): over a decade, HAS is expected to decline by 0.38 SD, consistent with physiological aging and the accumulation of impairments. Education exhibits strong, graded associations: high school completion is associated with a +0.294 SD, and college with a +0.534 SD, relative to up to primary. The college coefficient is large enough to offset the childhood-poverty penalty nearly fourfold, highlighting education as a potent lever of resilience. Current material conditions matter as well: being in the bottom wealth quartile is associated with a –0.255 SD, comparable to nearly two childhood-poverty penalties. Parental background retains an independent, albeit smaller, imprint: low parental occupational position associates with –0.050 SD after controlling for the individual's own education and wealth, supporting an intergenerational perspective in healthy aging.

In the presence of all these associations, childhood poverty remains significant, suggesting that life course shaping of childhood poverty operates beyond the pathway of socioeconomic positions into adulthood. Marital status shows positive associations for married/partnered (+0.153 SD) and, interestingly, for separated/widowed (+0.088 SD), which could reflect selective mortality; country controls attenuate unobserved heterogeneity. The fixed-effects model includes country dummies and explains a sizable share of variance (adjusted *R*^2^ = 0.283) for a composite, multidimensional outcome. The near-identical fixed- and random-effects estimates, alongside very large z-statistics, underscore the stability of the results across modeling choices and strengthen the conclusion that childhood poverty exerts an independent and consistent restraint on healthy aging worldwide.

### 3.3 Marginal plot for ages 70–90

**Figure 2:**
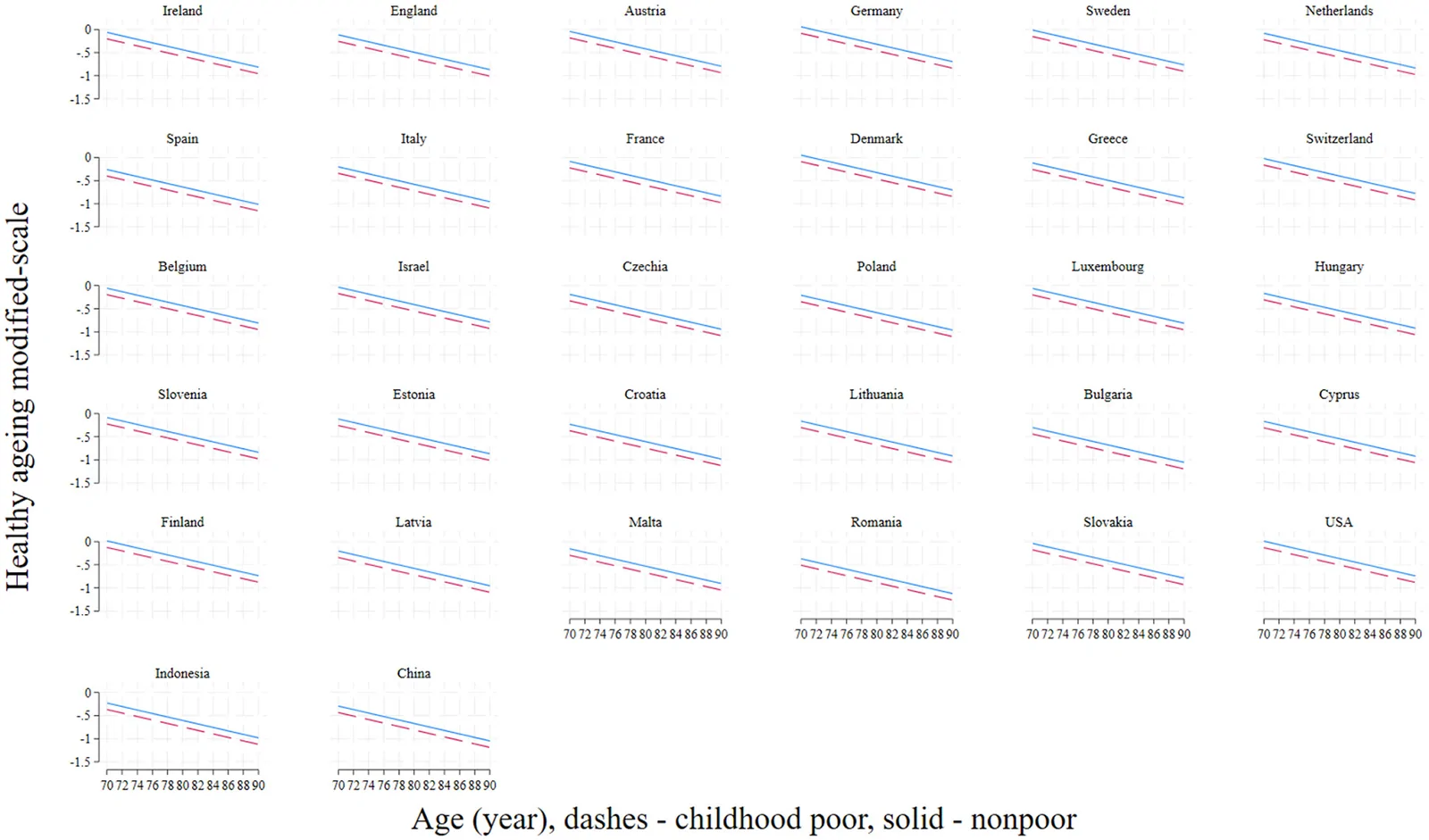
Marginal predictions of Healthy Aging Scale (modified) for ages 70–90 across 32 nations, dashed = childhood poor; solid = non-poor.

To ease comprehension, I draw in Figure 2 marginal predictions for ages 70—90, plotted for each country, showing that in all settings the childhood-poor age profiles (dashed lines) lie below the non-poor profile (solid lines) throughout. This trellis plot shows, at a glance, a pervasive childhood poverty penalty for healthy aging. Moreover, both baseline levels (age 70) and slopes vary widely across countries. In some nations, the gap is approximately parallel across ages (stable disadvantage); elsewhere, it widens (cumulative disadvantage). Occasional convergence at extreme ages likely reflects selective mortality or survival bias.

### 3.4 Supplementary analyses: model results for latent class construction of childhood poverty

Per prior literature, childhood poverty is a latent class (two classes: poor and non-poor) which is indicated by a set of items or indicators (e.g., overcrowding, lack of home facilities such as an indoor toilet). For each cohort, each latent class model result includes fit statistics (Akaike and Bayes-Schwarz Information Criteria), class probabilities, item-response probabilities, and classification uncertainty. The latent class construction gives a series of tables (Tables 5–10).

**Table 5:** Results of model estimation for childhood poverty—Europe (SHARE).

AIC	BIC	Classification error
366499.459	366632.267	0.067
Class probabilities		
	Poor = 0.24	Non-poor = 0.76
Item probabilities (poor/non-poor)		
Overcrowding: No	0.047	0.890
Overcrowding: Yes	0.953	0.110
Number of lacks: none	0.011	0.210
Number of lacks: 1	0.020	0.166
Number of lacks: 2	0.036	0.128
Number of lacks: 3	0.090	0.142
Number of lacks: 4	0.202	0.143
Number of lacks: 5	0.643	0.210
Number of books: none	0.651	0.308
≤ 10	0.223	0.236
*A shelf*	0.107	0.276
*A bookcase*	0.014	0.092
*≥ Two bookcases*	0.005	0.088

Facilities: running hot water, running cold water, indoor toilet, fixed bath, central heating.

**Table 6:** Results of model estimation for childhood poverty—Ireland (TILDA).

AIC	BIC	Classification error
14824.152	14950.512	0.02
Class probabilities		
	Poor = 0.92	Non-poor = 0.08
Item probabilities (poor/non-poor)		
Childhood finance: hardship	0.113	0.112
Childhood finance: neutral	0.690	0.608
Childhood finance: no hardship	0.197	0.279
Father worked: No	0.074	0.060
Father worked: Yes	0.926	0.940
Number of lacks: none	0.000	0.150
Number of lacks: 1	0.000	0.286
Number of lacks: 2	0.000	0.100
Number of lacks: 3	0.000	0.107
Number of lacks: 4	0.000	0.139
Number of lacks: 5	1.000	0.218
Number of books: none	0.998	0.048
≤ 10	0.002	0.358
*A shelf*	0.000	0.215
*A bookcase*	0.000	0.222
*Two bookcases*	0.000	0.072
*> Two bookcases*	0.000	0.086

Facilities: running hot water, running cold water, indoor toilet, fixed bath, central heating.

**Table 7:** Results of model estimation for childhood poverty—Indonesia (IFLS).

AIC	BIC	Classification error
48011.278	48026.278	0.084
Class probabilities		
	Poor = 0.58	Non-poor = 0.42
Item probabilities (poor/non-poor)		
Overcrowding: No	0.144	0.287
Overcrowding: Yes	0.856	0.713
Experienced hunger: No	0.861	0.950
Experienced hunger: Yes	0.139	0.050
Electricity: No	0.813	0.027
Electricity: Yes	0.187	0.973
Running cold water: No	0.976	0.635
Running cold water: Yes	0.024	0.365
Indoor toilet: No	0.884	0.227
Indoor toilet: Yes	0.116	0.773

**Table 8:** Results of model estimation for childhood poverty—United States (HRS).

AIC	BIC	Classification error
102298.892	102147.105	0.069
Class probabilities		
	Poor = 0.17	Non-poor = 0.83
Item probabilities (poor/non-poor)		
Overcrowding: No	0.679	0.690
Overcrowding: Yes	0.321	0.310
Number of lacks: none	0.011	0.210
Number of lacks: 1	0.020	0.166
Number of lacks: 2	0.036	0.128
Number of lacks: 3	0.090	0.142
Number of lacks: 4	0.202	0.143
Number of lacks: 5	0.643	0.210
Number of books: none	0.490	0.329
≤ 10	0.266	0.261
*A shelf*	0.182	0.261
*A bookcase*	0.040	0.085
*≥ Two bookcases*	0.021	0.065
Fostered: No	0.837	1.000
Fostered: Yes	0.163	0.000
Financial difficulty: No	0.877	0.912
Financial difficulty: Yes	0.123	0.088
Lived with grandparent: No	0.560	0.991
Lived with grandparent: Yes	0.440	0.009

Facilities: running hot water, running cold water, indoor toilet, fixed bath, central heating.

**Table 9:** Results of model estimation for childhood poverty—England (ELSA).

AIC	BIC	Classification error
51453.273	51349.396	0.070
Class probabilities		
	Poor = 0.18	Non-poor = 0.82
Item probabilities (poor/non-poor)		
Overcrowding: No	0.152	0.943
Overcrowding: Yes	0.848	0.057
Number of lacks: none	0.032	0.086
Number of lacks: 1	0.309	0.539
Number of lacks: 2	0.096	0.110
Number of lacks: 3	0.093	0.069
Number of lacks: 4	0.283	0.138
Number of lacks: 5	0.185	0.059
Number of books: none	0.533	0.252
≤ 10	0.251	0.233
*A shelf*	0.173	0.315
*A bookcase*	0.029	0.103
*≥ Two bookcases*	0.014	0.096

Facilities: running hot water, running cold water, indoor toilet, fixed bath, central heating.

**Table 10:** Results of model estimation for childhood poverty—China (CHARLS).

AIC	BIC	Classification error
135613.740	135439.372	0.023
Class probabilities		
	Poor = 0.09	Non-poor = 0.91
Item probabilities (poor/non-poor)		
Starved: No	0.271	0.249
Starved: Yes	0.729	0.751
Financial hardship: No	0.350	0.627
Financial hardship: Yes	0.650	0.373
Had to move: No	0.905	0.920
Had to move: Yes	0.095	0.080
Unsafe commune: No	0.441	0.918
Unsafe commune: Yes	0.559	0.082
Less willing to help commune: No	0.078	0.930
Less willing to help commune: Yes	0.922	0.070
Clean commune: Yes	0.282	0.653
Clean commune: No	0.718	0.347

Because the latent class construction of childhood poverty using TILDA appears problematic (class probabilities are out of line with those from the other cohorts), I instead left it out and re-ran the main analysis. The main results stand (Table 11).

**Table 11:** Five cohorts analysis, leaving out TILDA.

Exposure/control	Country fixed effects regression		Random intercepts regression	
	Estimate	(t)	Estimate	(t)
Childhood poor	–0.139***	(–16.81)	–0.140***	(–16.96)
Sex, women	–0.276***	(–43.49)	–0.276***	(–43.49)
Age	–0.038***	(–108.27)	–0.038***	(–108.30)
High school	0.299***	(38.31)	0.299***	(38.42)
College/more	0.535***	(57.34)	0.536***	(57.47)
Married/partnered	0.159***	(10.60)	0.158***	(10.57)
Separated/Widowed	0.099***	(6.41)	0.095***	(6.38)
Bottom quartile wealth	–0.256***	(–28.83)	–0.257***	(–29.08)
Parental occupational position low	–0.060***	(–7.08)	–0.061***	(–7.17)
Constant	2.419***	(82.27)	2.414***	(66.13)
Country dummies	Yes		No	
Observations	76,003		76,003	
Adjusted R^2^	0.286		0.272	

*t* statistics in parentheses; ^*^p < 0.1, ^**^p < 0.05, ^***^p < 0.01.

### 3.5 Supplementary sensitivity analyses: sex stratification, cohort-specific results, and sample non-response patterns

I assess the main results above against three alternatives: first, whether the results deviate considerably across the sexes; second, whether the results differ considerably across specific cohorts; and third, whether sample construction potentially affects the results. In other words, because not all participants agreed to give life history interviews, nor give all responses to healthy aging items, the analytic sample (made up of those who agreed to both) may be biased; say they tended to be younger—the non-response patterns need to be examined.

In Table 12, sex-stratified models confirm robustness: childhood poverty lowers healthy aging for both women (–0.150 SD) and men (–0.125 SD), with nearly identical age slopes (–0.038 per year), supporting a consistent life course penalty across sexes. Education remains protective (high school +0.298 women; +0.275 men; college +0.53 both), while bottom-quartile wealth is detrimental (women –0.264; men –0.249). Marital status benefits are larger for men (married/partnered +0.208) than women (+0.106), and parental low occupational position has small negative effects in both sexes (–0.05).

**Table 12:** Results of model estimation for healthy aging scale by sex.

Exposure/control	Women	Men
Childhood poor	−0.150^***^	−0.125^***^
	(−14.45)	(−10.17)
Age	−0.037^***^	−0.038^***^
	(−83.54)	(−71.14)
High school	0.298^***^	0.275^***^
	(29.68)	(23.87)
College/more	0.535^***^	0.525^***^
	(43.69)	(38.48)
Married/partnered	0.106^***^	0.208^***^
	(5.46)	(10.10)
Separated/widowed	0.038^**^	0.142^***^
	(1.97)	(6.21)
Wealth: bottom quartile	−0.264^***^	−0.249^***^
	(−23.79)	(−17.87)
Parental occupational position low	−0.052^***^	−0.048^***^
	(−4.94)	(−3.81)
Constant	2.188^***^	2.321^***^
	(57.45)	(55.21)
Observations	45,472	34,841
Adjusted *R*^2^	0.291	0.234

*t* statistics in parentheses; ^*^p < 0.1, ^**^p < 0.05, ^***^p < 0.01.

In Tables 13–18 series, cohort-specific estimates reaffirm the main result but also reveal meaningful heterogeneity. The childhood-poverty penalty is strongest in China/CHARLS (–0.311), sizeable in SHARE (–0.168), IFLS (–0.187), and HRS (–0.184), moderate in TILDA (–0.133), and smallest in ELSA (–0.063). Women's disadvantage is largest in CHARLS (–0.465), while the wealth penalty peaks in HRS (–0.489) and ELSA (–0.406) but is attenuated in IFLS (–0.094) and CHARLS (–0.148). Education returns are strongest in IFLS and CHARLS. Overall, findings are robust, with variations plausibly linked to history, welfare regimes and social development.

**Table 13:** Results of model estimation for healthy aging scale per cohort—Europe (SHARE).

Exposure/control	Europe (SHARE)
Childhood poor	−0.168^***^
	(−18.43)
Sex, women	−0.244^***^
	(−31.22)
Age	−0.042^***^
	(−97.20)
High school	0.291^***^
	(32.94)
College/more	0.550^***^
	(51.95)
Married/partnered	0.132^***^
	(7.54)
Separated/widowed	0.097^***^
	(5.15)
Wealth: bottom quartile	−0.285^***^
	(−28.10)
Parental occupational position low	−0.051^***^
	(−5.72)
Constant	2.679^***^
	(78.10)
Observations	49,880
Adjusted *R*^2^	0.302

*t* statistics in parentheses; ^*^p < 0.1, ^**^p < 0.05, ^***^p < 0.01.

**Table 14:** Results of model estimation for healthy aging scale per cohort—Ireland (TILDA).

Exposure/control	Ireland (TILDA)
Childhood poor	−0.133^***^
	(−4.54)
Sex, women	−0.267^***^
	(−11.39)
Age	−0.026^***^
	(−18.08)
High school	0.233^***^
	(7.94)
College/more	0.530^***^
	(13.78)
Married/partnered	0.146^***^
	(3.70)
Separated/widowed	−0.041
	(−0.90)
Wealth: bottom quartile	−0.166^***^
	(−3.98)
Parental occupational position low	0.039
	(1.64)
Constant	1.603^***^
	(15.06)
Observations	4,310
Adjusted *R*^2^	0.188

*t* statistics in parentheses; ^*^p < 0.1, ^**^p < 0.05, ^***^p < 0.01.

**Table 15:** Results of model estimation for healthy aging scale per cohort—England (ELSA).

Exposure/control	England (ELSA)
Childhood poor	−0.062^***^
	(−2.61)
Sex, women	−0.225^***^
	(−11.32)
Age	−0.031^***^
	(−28.48)
High school	0.238^***^
	(9.55)
College/more	0.425^***^
	(16.86)
Married/partnered	0.108^**^
	(2.56)
Separated/widowed	−0.016
	(−0.36)
Wealth: bottom quartile	−0.406^***^
	(−16.84)
Parental occupational position low	−0.159^***^
	(−3.63)
Constant	2.019^***^
	(23.26)
Observations	7,226
Adjusted *R*^2^	0.261

*t* statistics in parentheses; ^*^p < 0.1, ^**^p < 0.05, ^***^p < 0.01.

**Table 16:** Results of model estimation for healthy aging scale per cohort—US (HRS).

Exposure/control	US (HRS)
Childhood poor	−0.184^***^
	(−5.32)
Sex, women	−0.251^***^
	(−10.77)
Age	−0.025^***^
	(−22.39)
High school	0.059
	(1.52)
College/more	0.366^***^
	(9.52)
Married/partnered	0.145
	(1.20)
Separated/widowed	0.047
	(1.12)
Wealth: bottom quartile	−0.489^***^
	(−14.40)
Parental occupational position low	NA
Constant	1.731^***^
	(18.42)
Observations	6,386
Adjusted *R*^2^	0.158

*t* statistics in parentheses; ^*^p < 0.1, ^**^p < 0.05, ^***^p < 0.01. 99% of parental occupation is not released for public use.

**Table 17:** Results of model estimation for healthy aging scale per cohort—Indonesia (IFLS).

Exposure/control	Indonesia (IFLS)
Childhood poor	−0.187^***^
	(−3.92)
Sex, women	−0.248^***^
	(−7.55)
Age	−0.038^***^
	(−19.49)
High school	0.439^***^
	(12.09)
College/more	0.824^***^
	(15.26)
Married/partnered	0.332^**^
	(2.15)
Separated/widowed	0.344^**^
	(2.20)
Wealth: bottom quartile	−0.095^**^
	(−2.53)
Parental occupational position low	−0.059^*^
	(−1.96)
Constant	2.100^***^
	(10.54)
Observations	3,097
Adjusted *R*^2^	0.269

*t* statistics in parentheses; ^*^p < 0.1, ^**^p < 0.05, ^***^p < 0.01.

**Table 18:** Results of model estimation for healthy aging scale per cohort—China (CHARLS).

Exposure/control	China (CHARLS)
Childhood poor	−0.311^***^
	(−9.16)
Sex, women	−0.465^***^
	(−25.39)
Age	−0.035^***^
	(−28.71)
High school	0.473^***^
	(21.79)
College/more	0.806^***^
	(8.83)
Married/partnered	0.350^***^
	(3.27)
Sep/widowed	0.301^***^
	(2.74)
Bottom quartile	−0.148^***^
	(−7.38)
Parental occupational position low	−0.290^***^
	(−6.33)
Constant	2.148^***^
	(15.67)
Observations	9,414
Adjusted *R*^2^	0.278

*t* statistics in parentheses; ^*^p < 0.1, ^**^p < 0.05, ^***^p < 0.01.

### 3.6 Non-response patterns for retrospective interviews

The analytic sample comprises prospective participants who agreed to give life history interviews. Some declined, and there may be significant differences between the analytic vs. declined sample, tested using χ^2^ and *t* tests for nominal and continuous variables as appropriate (Table 19). In the HRS, the analytic sample has 59% women and 41% men, with $\chi_{1}^{2}=1.0$ and *p* value = 0.30; a pattern also observed in the sample who declined the interviews. Conversely, in other cohorts, women in the analytic samples are more likely to agree to the interviews. Meanwhile, in the HRS the analytic sample has older participants, and this difference is significant, while in ELSA the opposite is true and also significant. In sum, there are no strong patterns of participation in retrospective interviews worldwide.

**Table 19:** Non-response patterns across analytic vs. declined sample in HRS, ELSA, SHARE, TILDA, CHARLS, and IFLS.

Survey	Variable	Declined sample	Analytic sample	Test statistic/*p*-value
HRS	Women	60%	59%	$\chi_{1}^{2}=1.0$, *p* = 0.30
	Men	40%	41%	
	Mean age	65.1 year	68.4 year	*p* < 0.001
ELSA	Women	74%	55%	$\chi_{1}^{2}=87.8$, *p* < 0.001
	Men	26%	45%	
	Mean age	65.3 year	54.1 year	*p* < 0.001
SHARE	Women	56%	58%	$\chi_{1}^{2}=8.9$, *p* = 0.003
	Men	44%	42%	
	Mean age	67.3 year	68.5 year	*p* < 0.001
CHARLS	Women	48%	53%	$\chi_{1}^{2}=36.8$, *p* < 0.001
	Men	52%	47%	
	Mean age	62.2 year	64.1 year	*p* < 0.001
IFLS	Women	59%	53%	$\chi_{1}^{2}=19.0$, *p* < 0.001
	Men	41%	47%	
	Mean age	70.7 year	59.8 year	*p* < 0.001
TILDA	Women	89%	54%	$\chi_{1}^{2}=155$, *p* < 0.001
	Men	11%	46%	
	Mean age	49 year	63.5 year	*p* < 0.001

## 4 Discussion

This study advances the scholarship on healthy aging in several important respects. It provides some of the strongest and broadest empirical support for the positive view of healthy aging, not as the absence of disease but as the maintenance of functional ability across multiple dimensions. By demonstrating that childhood poverty exerts a persistent influence on healthy aging decades later, the study also shows that the diversity observed in older age is not simply a consequence of biological aging. Instead, healthy aging is shaped across the life course through the accumulation of advantages and disadvantages, especially at social origin or parental socioeconomic position, confirming the emerging understanding of the life course shaping of healthy aging. The results therefore stress the balance toward a developmental understanding of healthy aging as a lifelong process rooted in childhood conditions, education and working life, and socioeconomic environments.

The childhood poverty penalty provides a clear, positive-orientation analog to prior deficit-oriented outcomes: where earlier studies observed higher depression, greater probable sarcopenia, more frailty or more multimorbidity among those raised in poverty (13–15, 19), we now observe less healthy aging. The penalty is roughly half the sex gap and one-quarter of the college advantage. The robustness of this association to model specification and to sex-stratification underscores a widely shared life course imprint of material deprivation despite contemporary socioeconomic attainment, e.g., education and wealth. This is consistent with epigenetic evidence I supplied elsewhere showing that childhood poverty epigenetically accelerates aging (14).

Cross-country heterogeneity is informative rather than contradictory. Higher baselines and gentler declines -visible in several northern European settings- are consistent with comprehensive primary care, rehabilitation, and social care supports, while steeper slopes in other settings may reflect unmet need for chronic-disease management, limited rehabilitation coverage, or less friendly built environments for aging. The cohort-by-cohort estimates further suggest historically rooted differences: e.g., the strongest childhood poverty penalty in China plausibly aligns with cohort exposure to the Great Leap Forward and rapid social transition (58, 59) whereas smaller penalties in England may reflect longer-standing welfare protections (14, 15, 19, 60).

### 4.1 Advancing a faithful implementation of the WHO life course framework

This study provides one of the clearest presentations of the WHO life course framework for healthy aging–treating health in later life as the product of cumulative exposures, critical periods, and interacting social × biological pathways across the entire span from childhood to old age–while measuring the outcome as functional ability rather than mere disease absence (5). I first constructed a modified healthy aging scale as a latent trait that integrates cognition, locomotion, sensory function, vitality, activities of daily living and mental health; then modeled childhood poverty as a latent construct to capture material disadvantages highlighted in the WHO framework (housing quality, overcrowding, financial hardship) (5); further, examined associations after adjusting for parental socioeconomic position, age, sex, education, wealth, marital status and country dummies, thereby locating later-life functioning within a frame of structural determinants rather than individual risk factors alone.

In doing so, the study extends recent cross-country life course evidence on single domains such as episodic memory or grip strength to a positive, composite outcome in which higher scores mean healthier aging, fully aligned with WHO's emphasis on functionings (2, 5). The core result (a childhood poverty penalty on healthy aging) provides a coherent bridge from deficit-oriented outcomes to a capabilities-oriented metric of later-life functioning.

### 4.2 Breadth and generalizing beyond 32 nations

A second advance is geographic reach. By pooling six longitudinal aging cohorts—HRS (USA), ELSA (England), SHARE (27 European countries), TILDA (Ireland), CHARLS (China), IFLS (Indonesia)—I study the life course imprint of childhood poverty across 32 nations that differ widely in income levels, welfare regimes, epidemiological transitions and health systems (60). The country fixed effects regression and the trellis of marginal predictions for ages 70–90 jointly show that the childhood poverty penalty is ubiquitous: predicted HAS for the childhood poor (dashed) lies below the non-poor (solid) at the same age.

Yet the levels (baselines at 70) and slopes (declines to 90) vary substantially, reflecting differences in prevention, rehabilitation, social protection, cohort shocks and diagnostic capacity, exactly the heterogeneity the WHO framework expects. This combination (consistent penalty, heterogeneous trajectories) enhances external validity while cautioning against one-size-fits-all prescriptions. Moreover, I document meaningful cross-cohort variation in the magnitude of the penalty: strongest in China, moderate in Ireland, and smallest in England. These differences plausibly reflect cohort-specific histories (e.g., the Great Leap Forward legacy in China) (20, 59), social policy, and measurement contrast (e.g., wealth reporting, depression coverage) even after harmonization and fixed-effects controls. Such heterogeneity echoes earlier cross-country patterns reported for frailty, multimorbidity, cognitive functions, and probable sarcopenia (14, 15, 19), but now in the healthy aging scale (modified), which captures the net functional consequences of those domain-specific deficits.

### 4.3 Addressing the imbalance in the evidence base

A recurring limitation in the literature is that life course evidence is overrepresented in high-income Western settings; consequently, global health policies risk being extrapolated from contexts with dense data and strong health systems to those with sparse data and resource-constrained systems (45). Phrased differently, policies risk being extrapolated from places which grew rich before growing old to places which are growing old while not yet rich. This study directly reduces that imbalance by adding China and Indonesia to the set of trans-Atlantic nations and by reporting pooled and cohort-by-cohort estimates.

For example, educational gradients are steepest where schooling expanded later and faster (China and Indonesia), consistent with structural change in human capital formation; wealth penalties are large where financial protection is less universal (US), illustrating that current socio-economic conditions continue to shape functionings in later life. By placing these results alongside Europe and US findings, the study broadens the empirical canvas on which the WHO Decade of Healthy Aging (3) can paint.

### 4.4 Recall error: from problem recognition to methodological solution

Unlike most studies of childhood conditions and their association with older adults' health, this study is deliberately self-critical about retrospective childhood measures. It quantifies recall error using independent validations: in England, only one-in-three of 50- or 62-year-olds correctly recalled both the number of rooms and the number of people in their childhood vs. records collected from their mothers decades earlier. It is unsafe to treat childhood recollections as error-free. Consequently, following recent practices, childhood poverty is a latent construct measured using diverse indicators (e.g., overcrowding, facilities, financial hardship, famine/starvation). This practice is here brought to bear on healthy aging and across a wider set of countries, improving inference without discarding imperfect yet valuable data.

Beyond bias control, the latent construct approach enables comparability when indicator sets differ (e.g., ELSA/SHARE ask about running hot water and central heating; HRS emphasizes financial events; CHARLS includes death from starvation). By targeting the association while controlling for country effects, I can compare strength and direction of life course links even if prevalence is not directly harmonized-the argument used in cross-national studies of depression and allostatic load (29, 30). The novel step here is to apply that argument to a modified healthy aging scale, preserving metric information via a generalized latent trait model rather than dichotomizing continuous items, thereby aligning statistical practice with the conceptual complexity of healthy aging.

### 4.5 From “long arm” mechanisms to “healthy aging” consequences

I have proposed and supported biological pathways -notably epigenetic aging measured by methylation clocks- to explain how childhood poverty “gets under the skin” (14). Here I extend the consequences of those pathways to a composite functional outcome: the same early-life hardship that predicted higher depression, worse muscle function, frailty or multimorbidity now predicts lower healthy aging decades later, even after accounting for parental and personal socioeconomic positions.

This coherence across domains strengthens the plausibility of life course mechanisms while remaining cautious about causal claims. The convergence of epidemiologic (childhood poverty penalty), socio-economic (education/wealth gradients) and biological (epigenetic age) signals is noteworthy: a single, conceptually unified story can now be told about how childhood conditions become embedded and what that embedding looks like when the outcome is functionings in later life.

### 4.6 Strengths and limitations

This study is subject to several limitations which warrant careful consideration. First, the analytical framework employed herein is relatively simple and does not constitute a full structural model (17). While precedents for such simplified approaches exist (13, 14, 61), the structural alternative has led some researchers to posit that including additional covariates might attenuate the observed influence of childhood conditions. This proposition remains an eminently empirical question. Nonetheless, the imperative persists: a more comprehensive framework should be pursued when harmonized data on youth and adult conditions across wealthy and developing nations become available.

Second, the study's focus on material deprivation in childhood excludes psychological dimensions, such as parent-child bonding relationships. These aspects are central to child development and represent a distinct stage within the broader life course framework. Their omission constitutes a notable limitation. Third, the inclusion of country fixed effects serves to control for unobserved heterogeneity across national contexts, including historical trajectories and health systems. Although this approach follows precedent (13, 14, 17), it may be deemed insufficient. Accordingly, country-specific analyses for the 32 nations in this study are deferred to a future monograph.

Fourth, consistent with the life course framework, this work is not a direct causal proof that eliminating childhood poverty will necessarily improve healthy aging decades later. Elsewhere, I noted that the absence of random assignment to childhood material conditions introduces potential confounding, as individuals who experienced childhood poverty may differ systematically from those who did not (14, 15). Moreover, survivor bias may be present, as some individuals exposed to childhood poverty may not have lived to participate in aging surveys. While this bias may suggest a directional effect, its magnitude remains unknown. Furthermore, the feasibility of randomized designs in this context is questionable, given the extended temporal gap -often spanning half a century- between exposure and outcome. Such a duration increases the likelihood of behavioral adaptation following randomization, thereby undermining causal inference. Despite these constraints, support for the life course framework may be bolstered through cross-national design.

Fifth, I acknowledge that the set of items or indicators of childhood conditions varies across cohorts; I would have preferred a uniform set. Balanced against this is the affordance of large-scale cross-national study even if the sets are not uniform. Though preferably they are, latitude may raise caution: lack of running hot water rightly indicates material deprivation in sub-arctic latitude; less so at the equator.

Sixth, the latent approach here and elsewhere (16, 17) mitigates but does not eliminate measurement error. As I discussed in the same article that motivates this work, there is the possibility of differential recall by current health status (13). In fact, Brown gave an example using the British 1958 cohort, known as the National Child Development Study, in which asthmatic 50-year-olds reported less overcrowded childhoods (62). This possibility can be explored in future work. Last, a twofold parental occupation (low, high) is too coarse, though it has precedents as a control in recent literature which span the wealthy and developing nations (25, 52).

The strengths of this study merit emphasis. First, faithful implementation of the WHO life course. The analysis aligns measurement (modified HAS), key exposure (latent childhood poverty with indicators of overcrowding and housing qualities), and covariate structure (parental occupational position, age, sex, education, wealth, marital status, country fixed effects) with the WHO framework's emphasis on functional ability and social determinants, avoiding a disease-only lens. Second, an exceptional geographic scope for the key exposure. Six cohorts across 32 nations provide both breadth and heterogeneity, giving confidence in generalization and revealing country variation (levels and slopes) in later-life functioning. Third, methodological discipline on recall error. There is scant evidence on recall error in retrospective information, and when evidence is found at age 50 and beyond, it is sizeable—two-thirds got childhood conditions wrong. This is addressed with latent constructs and corrected standard errors, improving validity without discarding data. Fourth, an outcome is constructed by preserving indicators' metrics. Using a generalized latent trait model retains metric information and suits multinational analyses where item distributions differ, aligning methodology with conceptual nuance. Last, robustness. Fixed vs. random effects yield nearly identical coefficients; sex-stratified and cohort-specific models confirm the childhood poverty penalty and the protective role of education, all supporting robustness.

### 4.7 Conclusion by way of informing global health policy

The childhood poverty penalty for healthy aging, together with its strong education and wealth gradients, indicates that later-life functionings are markedly shaped by early-life material conditions and remain malleable through current socioeconomic support. National plans under the UN Decade of Healthy Aging should embed child poverty reduction -better housing quality or an adequate number of rooms, for example- within aging policies, not just within child or social policies. Then, balance early- and mid-life investment. Countries with parallel healthy aging scale (modified) age profiles for the poor vs non-poor should prioritize closing the initial gap (family support, early learning support, housing improvements). Where widening gaps mark the age profiles, complement early action with midlife interventions: chronic disease management, age-friendly environments, and financial protection to buffer the wealth penalty observed here.

Further, target educational returns where they are largest. The considerable college advantage in China and Indonesia suggests that human capital expansion can substantially raise later-life functional capacity in rapidly developing settings; conversely, sustained adult learning programs may help sustain healthy aging in high-income societies. On a more practical level, employ measurement more widely. Routine incorporation of life history interviews, latent indicators of childhood conditions, and metric-preserving healthy-aging scale and its items can turn retrospective data into policy-relevant signals while avoiding unsafe inference.

More ambitiously, enriching biomarker collections with methylation (as in the HRS Venous blood sample I used to show that the childhood poor age epigenetically faster) (14) and neuropathological biomarkers (63) allows nations to track mechanistic links that inform prevention and rehabilitation. Lastly, use such measurements and evidence to plan for long-term or social care labor. The trellis plot showing between-country levels/slopes implies very different future demands of functional support. Wealthy and developing nations should anticipate long-term care demand and develop sustainable care-worker policies (training and retention) to avoid zero-sum competition as population aging accelerates worldwide.

Healthy aging is a life course achievement: to raise the healthy aging scale (modified) fairly and efficiently, reduce childhood poverty, support older adults' material security, and sustain functional capacity through prevention, rehabilitation, and supportive environments across the arc of life.

## Data Availability

Publicly available datasets were analyzed in this study. This data can be found here: HRS https://hrs.isr.umich.edu, SHARE www.share-project.org, ELSA www.elsa-project.ac.uk, CHARLS https://charls.pku.edu.cn, IFLS https://www.rand.org/well-being/social-and-behavioral-policy/data/FLS/IFLS.html, TILDA https://tilda.tcd.ie.

## References

[1] World Health Organization. World Report on Ageing and Health. World Health Organization (2015). Available online at: https://www.who.int/publications/i/item/9789241565042 (Accessed August 20, 2025).

[2] World Health Organization. Decade of Healthy Ageing: Baseline Report. World Health Organization (2020). Available online at: https://www.who.int/publications/i/item/9789240017900 (Accessed August 20, 2025).

[3] World Health Organization. UN Decade of Healthy Ageing 2021-2030 (2021). Available online at: https://www.who.int/initiatives/decade-of-healthy-ageing (Accessed August 20, 2025).

[4] Beard JR Officer A de Carvalho IA Sadana R Pot AM Michel JP . The World report on ageing and health: a policy framework for healthy ageing. Lancet. (2016) 387:2145–54. doi: 10.1016/S0140-6736(15)00516-4 26520231 PMC4848186

[5] World Health Organization. Framework to Implement a Life Course Approach in Practice. World Health Organization (2025). Available online at: https://www.who.int/publications/i/item/9789240112575 (Accessed August 20, 2025).

[6] Sanchez-Niubo A. Egea-Cortés L, Olaya B, Villalonga-Olives E, Caballero FF, Ayuso-Mateos JL, et al. Development of a common scale for measuring healthy ageing across the world: results from the ATHLOS consortium. Int J Epidemiol. (2021) 50:880–92. doi: 10.1093/ije/dyaa236 33274372 PMC8271194

[7] Lu W Pikhart H Sacker A. Comparing socio-economic inequalities in healthy ageing in the United States of America, England, China and Japan: evidence from four longitudinal studies of ageing. Ageing Soc. (2021) 41:1495–520. doi: 10.1017/S0144686X19001740

[8] Tampubolon G. Trajectories of the healthy ageing phenotype among middle-aged and older Britons, 2004-2013. Maturitas. (2016) 88:9–15. doi: 10.1016/j.maturitas.2016.03.002 27105690 PMC4850932

[9] Beard JR de Carvalho IA Sumi Y Officer A Thiyagarajan JA. Healthy ageing: moving forward. Bull World Health Organ. (2017) 95:730–730A. doi: 10.2471/BLT.17.203745 29147049 PMC5677619

[10] Kuh D Ben-Shlomo Y Lynch J Hallqvist J Power C. Life course epidemiology. J Epidemiol Community Health. (2003) 57:778–83. doi: 10.1136/jech.57.10.778 14573579 PMC1732305

[11] Kuh D Ben-Shlomo Y editors. A Life Course Approach to Chronic Disease Epidemiology. 2nd ed. Oxford: Oxford University Press (2004). doi: 10.1093/acprof:oso/9780198578154.001.0001

[12] Tampubolon G. Recall Error and Recall Bias in Life Course Epidemiology. Munich Personal RePEc Archive (MPRA) (2010). 23847. Available online at: https://mpra.ub.uni-muenchen.de/23847/ (Accessed August 20, 2025).

[13] Tampubolon G. Growing up in poverty, growing old in infirmity: the long arm of childhood conditions in Great Britain. PLoS ONE. (2015) 10:e0144722. doi: 10.1371/journal.pone.0144722 26675009 PMC4682716

[14] Tampubolon G. The long arm hypothesis: childhood poverty, epigenetic ageing, and late-life health in America, Britain, and Europe. In:Hoffmann R, editor. Handbook of Health Inequalities across the Life Course. Cheltenham: Edward Elgar Publishing (2023). p. 252–74. doi: 10.4337/9781800888166.00026

[15] Tampubolon G. Growing up in poverty, growing old in frailty: the life course shaping of health in the United States, England and Europe-a prospective and retrospective study. Sci Rep. (2025) 15:15510. doi: 10.1038/s41598-025-99929-2 40319157 PMC12049476

[16] Lennartsson C Eyjólfsdóttir HS Celeste RK Fritzell J. Social class and infirmity The role of social class over the life-course. SSM—*Popul Health*. (2018) 4:169–77. doi: 10.1016/j.ssmph.2017.12.001 29854902 PMC5976854

[17] Pakpahan E Hoffmann R Kröger H. The long arm of childhood circumstances on health in old age: evidence from SHARELIFE. Adv Life Course Res. (2017) 31:1–10. doi: 10.1016/j.alcr.2016.10.003

[18] Livingston G Huntley J Sommerlad A Ames D Ballard C Banerjee S . Dementia prevention, intervention, and care: 2024 report of the Lancet Standing Commission. Lancet. (2024) 404:572–628. doi: 10.1016/S0140-6736(24)01296-0 39096926

[19] Tampubolon G. Growing up in poverty, growing old with multimorbidity in America, Britain, China, Europe and Indonesia: a retrospective and prospective study. In:Asiamah N Khan H Nesser W Alvarez E, editors. Morbidity Prevention in Ageing. Cambrdige MA: Elsevier (2026). p. 100–21.

[20] Tampubolon G Li G. Early life adversity and late life dementia in the Harmonised Cognitive Assessment Protocol network (U.S., China, England and Europe). bioRxiv Preprint. (2025). doi: 10.1101/2025.06.04.25328976 40502612 PMC12155006

[21] Widagdo J Anggono V. The m^6^A-epitranscriptomic signature in neurobiology: from neurodevelopment to brain plasticity. J Neurochem. (2018) 147:137–52. doi: 10.1111/jnc.14481 29873074

[22] UCL Centre for Longitudinal Study. National Child Development Study; (2024).

[23] Tampubolon G. Intergeneration and Intrageneration Social Mobility in Britain. Manchester: University of Manchester (2009).

[24] Tampubolon G. Social Mobility and Long-Term Episodic Memory in Britain (2026). Under review at Scientific Reports. Preprint. doi: 10.64898/2026.04.12.26350709

[25] Wu YT Sanchez-Niubo A Caballero FF Prina M Ayuso-Mateos JL Haro JM . Childhood socioeconomic position and healthy ageing: results from five harmonised cohort studies in the ATHLOS consortium. BMJ Public Health. (2025) 3:e001590. doi: 10.1136/bmjph-2024-001590 40017978 PMC11865732

[26] Lara J Cooper R Nissan J Ginty AT Khaw KT Deary IJ . Towards measurement of the Healthy Ageing Phenotype in lifestyle-based intervention studies. Maturitas. (2013) 76:189–99. doi: 10.1016/j.maturitas.2013.07.007 23932426

[27] Birnbaum AL. Some latent trait models. In:Lord FM Novick MR, editors. Statistical Theories of Mental Test Scores. Reading MA: Addison-Wesley (1968). p. 397–479.

[28] Moustaki I Knott M. Generalized latent trait models. Psychometrika. (2000) 65:391–411. doi: 10.1007/BF02296153

[29] Tampubolon G Maharani A. When did old age stop being depressing? Depression trajectories of older Americans and Britons 2002-2012. Am J Geriatr Psychiatry. (2017) 25:1187–95. doi: 10.1016/j.jagp.2017.06.006 28734770 PMC5667578

[30] Courtin E Knapp M Grundy E Avendano-Pabon M. Are different measures of depressive symptoms in old age comparable? An analysis of the CES-D and Euro-D scales in 13 countries. Int J Methods Psychiatr. Res. (2015) 24:287–304. doi: 10.1002/mpr.1489 26314384 PMC6878602

[31] Tampubolon G Maharani A. Trajectories of allostatic load among older Americans and Britons: longitudinal cohort studies. BMC Geriatr. (2018) 18:255. doi: 10.1186/s12877-018-0947-4 30352552 PMC6199736

[32] Manly JJ Jones RN Langa KM Ryan LH Levine DA McCammon R . Estimating the prevalence of dementia and mild cognitive impairment in the US: the 2016 Health and Retirement Study Harmonized Cognitive Assessment Protocol Project. JAMA Neurol. (2022) 79:1242–9. doi: 10.1001/jamaneurol.2022.3543 36279130 PMC9593315

[33] Jones RN Manly JJ Langa KM Ryan LH Levine DA McCammon R . Factor structure of the Harmonized Cognitive Assessment Protocol neuropsychological battery in the Health and Retirement Study. J Int Neuropsychol Soc. (2024) 30:47–55. doi: 10.1017/S135561772300019X 37448351 PMC10787803

[34] Cohen J. The cost of dichotomization. Appl Psychol Meas. (1983) 7:249–53. doi: 10.1177/014662168300700301

[35] Altman DG Royston P. The cost of dichotomising continuous variables. BMJ. (2006) 332:1080. doi: 10.1136/bmj.332.7549.1080 16675816 PMC1458573

[36] MacCallum RC Zhang S Preacher KJ Rucker DD. On the practice of dichotomization of quantitative variables. Psychol Methods. (2002) 7:19–40. doi: 10.1037/1082-989X.7.1.19 11928888

[37] Greenland S. Avoiding power loss associated with categorization and ordinal scores in dose-response and trend analysis. Epidemiology. (1995) 6:450–4. doi: 10.1097/00001648-199507000-00025 7548361

[38] Cesari M Sadana R Sumi Y Thiyagarajan JA Banerjee A. What is intrinsic capacity and why should nutrition be included in the vitality domain? J Gerontol Ser A. (2022) 77:91–3. doi: 10.1093/gerona/glab318 35015816

[39] Sonnega A Faul JD Ofstedal MB Langa KM Phillips JWR Weir DR. Cohort Profile: the Health and Retirement Study (HRS). Int J Epidemiol. (2014) 43:576–85. doi: 10.1093/ije/dyu067 24671021 PMC3997380

[40] Steptoe A Breeze E Banks J Nazroo J. Cohort profile: the English longitudinal study of ageing. Int J Epidemiol. (2013) 42:1640–8. doi: 10.1093/ije/dys168 23143611 PMC3900867

[41] Börsch-Supan A Brandt M Hunkler C Kneip T Korbmacher J Malter F . Data resource profile: the Survey of Health, Ageing and Retirement in Europe (SHARE). Int J Epidemiol. (2013) 42:992–1001. doi: 10.1093/ije/dyt088 23778574 PMC3780997

[42] Donoghue OA McGarrigle CA Foley M Fagan A Meaney J Kenny RA. Cohort profile update: The Irish Longitudinal Study on Ageing (TILDA). Int J Epidemiol. (2018) 47:1398–1398l. doi: 10.1093/ije/dyy163 30124849

[43] Zhao Y Hu Y Smith JP Strauss J Yang G. Cohort profile: the China Health and Retirement Longitudinal Study (CHARLS). Int J Epidemiol. (2014) 43:61–8. doi: 10.1093/ije/dys203 23243115 PMC3937970

[44] Strauss J Witoelar F. Indonesia family life survey. In: Encyclopedia of Gerontology and Population Aging. Cham: Springer (2021). p. 2600–5. doi: 10.1007/978-3-030-22009-9_339

[45] Tampubolon G Maggang E. Evidence and Experience: UN Decade of Healthy Ageing around the World. Global Development Institute Blog (2025). Available online at: https://blog.gdi.manchester.ac.uk/evidence-and-experience-un-decade-of-healthy-ageing-around-the-world/ (Accessed August 20, 2025).

[46] Vable AM Gilsanz P Kawachi I. Is it possible to overcome the ‘long arm' of childhood socioeconomic disadvantage through upward socioeconomic mobility? J Public Health. (2019) 41:566–74. doi: 10.1093/pubmed/fdz018 30811528 PMC7967879

[47] Tampubolon G Fajarini M. The strong grip of childhood conditions in older Europeans. bioRxiv Preprint. (2018). doi: 10.1101/267526

[48] Goodman LA. Exploratory latent structure analysis using both identifiable and unidentifiable models. Biometrika. (1974) 61:215–31. doi: 10.1093/biomet/61.2.215

[49] Vermunt JK. Log-Linear Models for Event Histories vol 8 of Advanced Quantitative Techniques in the Social Sciences Series. Thousand Oaks, CA: Sage Publications (1997).

[50] Tampubolon G. Revisiting omnivores in America circa 1990s: the exclusiveness of omnivores? Poetics. (2008) 36:243–64. doi: 10.1016/j.poetic.2008.02.007

[51] Tampubolon G. Heterogeneity of cognitive ageing in older Britons: latent class trajectories of cognitive scores in ELSA 2002-2013. Eur Geriatr Med. 6(Suppl. 1):S22. doi: 10.1016/S1878-7649(15)30072-3

[52] Kröger H Fritzell J Hoffmann R. The association of levels of and decline in grip strength in old age with trajectories of life course occupational position. PLoS ONE. (2016) 11:e0155954. doi: 10.1371/journal.pone.0155954 27232696 PMC4883757

[53] Tampubolon G. Cognitive ageing in Great Britain in the new century: Cohort differences in episodic memory. PLoS ONE. (2015) 10:e0144907. doi: 10.1371/journal.pone.0144907 26713627 PMC4699214

[54] Tampubolon G. Delineating the third age: joint models of older people's quality of life and attrition in Britain 2002-2010. Aging Mental Health. (2015) 19:576–83. doi: 10.1080/13607863.2014.1003279 25642833 PMC4445379

[55] Vermunt JK Magidson J. LG-Syntax User's Guide version 6.1. Tilburg (NL): Statistical Innovations Europe (2025).

[56] Vermunt JK. Latent class modeling with covariates: two improved three-step approaches. Polit Anal. (2010) 18:450–69. doi: 10.1093/pan/mpq025

[57] Vermunt JK. Stepwise estimation of latent variable models: an overview of approaches. Stat Modell. (2025) 25:530–51. doi: 10.1177/1471082X251355693

[58] Knight J Ding S. China's Remarkable Economic Growth. Oxford: Oxford University Press (2012). doi: 10.1093/acprof:oso/9780199698691.001.0001

[59] Li Y Xue QL Odden MC Chen X Wu C. Linking early life risk factors to frailty in old age: evidence from the China Health and Retirement Longitudinal Study. Age Ageing. (2020) 49:208–17. doi: 10.1093/ageing/afz160 31957780 PMC7047816

[60] Britnell M. In Search of the Perfect Health System. London: Palgrave Macmillan (2015). doi: 10.1007/978-1-137-49662-1

[61] Case A Fertig A Paxson C. The lasting impact of childhood health and circumstance. J Health Econ. (2005) 24:365–89. doi: 10.1016/j.jhealeco.2004.09.008 15721050

[62] Brown M. Assessing recall of early life circumstances: evidence from the National Child Development Study. Longit Life Course Stud. (2014) 5:64–78. doi: 10.14301/llcs.v5i1.232

[63] Börsch-Supan M Börsch-Supan A Andersen-Ranberg K Borbye-Lorenzen N Cofferen J Deza-Lougovski Y . Cohort Profile Update: Survey of Health, Ageing and Retirement in Europe-Biomarker Data for Age-Related Health Conditions. Berlin: Munich Research Institute for the Economics of Aging and SHARE Analyses (2026). doi: 10.64898/2026.01.28.26344911 42709687

